# Legal Notes for Institutional Workers

**Published:** 1911-12-02

**Authors:** 


### LEGAL NOTES FOR INSTITUTIONAL WORKERS.

(The Editor will be pleased to answer Legal queries in this column.)

### Voluntary Hospitals and Compulsory Insurance.

The time is rapidly approaching when the Insurance Bill will pass from the hands of the legislator to the Statute-book. That every man is presumed to know the law is a quaint conceit of English jurisprudence; that the man on a 'bus should know the contents of this extraordinary measure is (to put it mildly) a wild presumption. Before the doors of suggestion and amendment are closed for ever, a mere lawyer would essay to explain in as simple language as possible that part of the Bill which most nearly affects the institutional worker. Criticism of the Bill, and suggestions for its alteration so as to improve the position of the hospital must be left to others.

### The Text of Clause 12.

The one clause which is supposed to benefit the voluntary hospital is that to which the marginal note is "Provisions in the case of contributors who are inmates of hospitals, etc." Reading it shortly, it is as follows: "No payment . . . shall be made on account of sickness, disablement, or maternity benefit . . . to or in respect of any insured person during any period when the person . . . is an inmate of any . . . hospital . . . or infirmary . . . supported . . . by a charity or voluntary subscriptions."

Pausing there, before considering the rest of the clause, it is as if the Legislature said: "If an insured person is fortunate enough to obtain hospital relief the State need not trouble about him. The money he and his employer have subscribed can be devoted to more deserving objects." Let us see whether the remainder of the clause relieves the hospital of the hardship to which it is subjected by the first part.

"During such period . . . the sum . . . shall be paid in whole or in part for the relief . . . or maintenance of his dependents (if any) in such manner as the society or committee . . . think fit; . . . or if such person, being a member of an approved society . . . and has no dependents, shall if an agreement for the purpose has been made between the society or committee, and the hospital . . . be paid in whole or in part . . . towards the maintenance of such person in the hospital." A proviso to the clause allows the maternity benefit in some cases to be paid to a hospital. "Dependents" within the meaning of the clause means such persons as the approved society or local Insurance Committee shall ascertain to be wholly or in part dependent upon the earnings of such inmate of any such institution as aforesaid.

### The True Significance of the Clause.

Even when written in the shortened form above adopted this clause is not altogether easy to understand. But three startling truths stand clearly revealed. In the first place, there can be no contribution to the hospital if the insured person has anyone dependent upon him. A case might occur in which 5s. a week would be amply sufficient when added to his or her other means of subsistence, to support a dependent while the worker was in hospital; yet not one penny piece can be allocated to the support of the institution, although the cost of hospital treatment may have to be reckoned in pounds, not shillings.

It seems that that part of the money which is not required by the dependents is to be swept into the Treasury net.

### The Nature of the Agreement.

The next point, which is clear as daylight, is that there can be no direct contribution to the hospital unless by agreement—not between the hospital and the insured person but between the hospital and the society or committee. Of course, any insured person may enter a hospital as a paying patient if so minded; but what is the agreement contemplated?

Is a new agreement to be made in every case and for every patient? If so, it must apparently be made when the insured person is in hospital, and in that case the committee or society will have the hospital authorities in a cleft stick. They may offer to pay or not as they choose; the hospital is not going to turn the patient into the street. On the other hand, if an agreement of a general nature is to be made beforehand, what form will it assume?

Is it to bind the hospital to take in all the insured persons in a district, at so much per head, whenever they happen to need hospital treatment? It cannot be so worded, because the committee could not subscribe to the hospital in respect of a person having dependents!

It is the vagueness of the Bill on points such as these that justify that feeling of apprehension in the institutional mind.

### The Deposit Contributor.

The last point which it is desired to emphasise is that there is no provision whereunder anything can be paid to a hospital direct in respect of the deposit or Post Office contributor. He may draw his full 10s. a week whether he is in hospital or not; whether he has dependents or not; and need pay nothing to the institution.

One may search the Bill from end to end without finding anything to modify the rigour of these provisions. Nor is it competent for a hospital to enter into an arrangement by which the money accruing due to an insured person shall be paid over.

The Bill provides that every assignment and every agreement to assign or charge any of the benefits conferred by the Act is void.

